# OrthoQuantum: visualizing evolutionary repertoire of eukaryotic proteins

**DOI:** 10.1093/nar/gkac385

**Published:** 2022-05-24

**Authors:** Ivan S Ilnitskiy, Anastasia A Zharikova, Andrey A Mironov

**Affiliations:** Faculty of Bioengineering and Bioinformatics, Lomonosov Moscow State University, Lomonosovsky Prospect 27, Building 10, 119991 Moscow, Russia; Kharkevich Institute of Information Transmission Problems, Russian Academy of Sciences, Big Karetny Lane 19, Building 1, 127051 Moscow, Russia; Faculty of Bioengineering and Bioinformatics, Lomonosov Moscow State University, Lomonosovsky Prospect 27, Building 10, 119991 Moscow, Russia; Kharkevich Institute of Information Transmission Problems, Russian Academy of Sciences, Big Karetny Lane 19, Building 1, 127051 Moscow, Russia; Faculty of Bioengineering and Bioinformatics, Lomonosov Moscow State University, Lomonosovsky Prospect 27, Building 10, 119991 Moscow, Russia; Kharkevich Institute of Information Transmission Problems, Russian Academy of Sciences, Big Karetny Lane 19, Building 1, 127051 Moscow, Russia

## Abstract

Extensive amounts of data from next-generation sequencing and omics studies have led to the accumulation of information that provides insight into the evolutionary landscape of related proteins. Here, we present OrthoQuantum, a web server that allows for time-efficient analysis and visualization of phylogenetic profiles of any set of eukaryotic proteins. It is a simple-to-use tool capable of searching large input sets of proteins. Using data from open source databases of orthologous sequences in a wide range of taxonomic groups, it enables users to assess coupled evolutionary patterns and helps define lineage-specific innovations. The web interface allows to perform queries with gene names and UniProt identifiers in different phylogenetic clades and supplement presence with an additional BLAST search. The conservation patterns of proteins are coded as binary vectors, i.e., strings that encode the presence or absence of orthologous proteins in other genomes. These strings are used to calculate top-scoring correlation pairs needed for finding co-inherited proteins which are simultaneously present or simultaneously absent in specific lineages. Profiles are visualized in combination with phylogenetic trees in a JavaScript-based interface. The OrthoQuantum v1.0 web server is freely available at http://orthoq.bioinf.fbb.msu.ru along with documentation and tutorial.

## Graphical Abstract

**Graphical Abstract:**
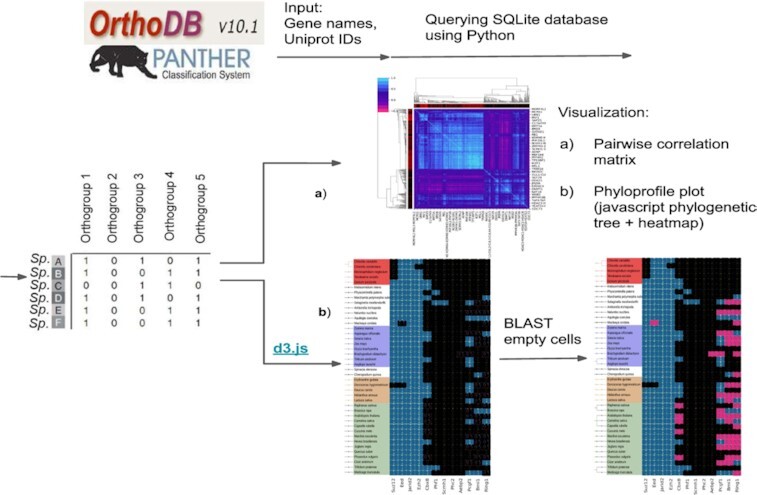
OrthoQuantum Web-application.

## INTRODUCTION

Conserved homologous sequences can be used to infer evolutionary history. The concepts of orthology and paralogy originated in the field of molecular taxonomy (1). Orthologs usually have the same function in different species, while paralogs tend to evolve towards functional diversification (2).

Knowing which homologous genes are orthologs and which are paralogs is necessary to build evolutionary scenarios that include vertical inheritance, horizontal transfer, loss, and specific gene duplications. There are several resources that define orthologous sequences. One of the first large-scale databases is COG (3) and its eukaryotic equivalent KOG. COG was the first to apply sequence comparison methods to predict clusters of orthologous proteins. Automated orthology prediction requires more sophisticated algorithms, such as the Inparanoid algorithms (4). InParanoid is a fully automatic method for finding orthologs and paralogs between two species and is especially useful for identifying inparalogs and outparalogs in eukaryotes. Unfortunately, InParanoid cannot process multiple genomes because it targets pairwise orthologous relationships and only analyzes two genomes at a time. OrthoDB has hierarchical classification of orthology: the clustering procedure is carried out separately for the main nodes of the evolutionary tree (5). Because more than two species have been investigated for protein orthology, there are several speciation events. In this case, an orthologous group refers to all descendants of an ancestral gene, which was the last common ancestor of the selected species.

Originally, phylogenetic profile or phyletic pattern studies used binary vectors, which indicate in which species the homolog is present or absent, to correlate gene occurrence (6,7). During the course of evolution, functional groups of proteins can disappear entirely in individual taxa or emerge as a result of gene duplications (8,9). The idea is that functionally linked genes are acquired and lost together, which leads to the correlation of the vectors. Binary vectors have been mostly implemented in predicting linkages between proteins but are also used to find other evolutionary events. A gene can also be replaced during evolution with another gene with identical or similar functions. This non-orthologous substitution (10) results in an anti-correlation of the occurrence profiles of the displaced and substituted genes. The phylogenetic profiling method yielded outstanding results in evolutionary studies of a wide range of prokaryotic and eukaryotic organelles such as cilia (11) and mitochondria (12). Recent attempts at analyzing phyletic patterns and detecting modules with shared evolutionary history employ advanced statistical modeling of correlated gene evolution (13,14). A range of databases and tools for visualization of Eukarya’s phylogenetic profiles have been developed (15–18).

OrthoQuantum is distinguished from other web servers by a number of features. The web server relies on orthology predictions from the OrthoDB database, which is leading in coverage of eukaryotic species, with 1226 species that have a complete or nearly complete genome assembly and 37 million genes/proteins in the most recent update. The use of well-annotated genomes is important to ensure that genes identified as ‘missing’ are not actually in the genome. For large taxonomic groups there is an option in our tool to continue with a compact set of species with a good quality of genome assembly, or with a full set of species from OrthoDB that may provide better resolution for conservation patterns (for example, the choice between 120 or 1200 species of eukaryotes). We also use PantherDB family/subfamily classification of homologous sequences (19). Genes/proteins are sorted based on their phylogenetic profiles using hierarchical clustering. These clusters are then presented in combination with phylogenetic trees using a JavaScript-based interface, which allows for quick identification of genes that are unique to distinct lineages. As a result, regions with different profiles of protein coverage become visible.

## MATERIALS AND METHODS

### Implementation and workflow

OrthoQuantum visualization is implemented using the Python Flask framework and JavaScript in HTML5. Apache is used as a reverse proxy server that forwards requests to a Docker container with multiple Gunicorn WSGI server instances that run Flask+Dash application responsible for providing UI. On the client side Dash uses React components from dash and dash-bootstrap-components libraries, as well as a custom React component that adapts PhyD3 javascript library to Dash framework. We used the PhyD3 package (20) for visualization of the phylogenetic profiles with species trees.

The management of computational workflows and batch jobs depends on a custom asyncio-based scheduler, which is supported by the Redis message broker. The data from OrthoDB and PantherDB have been organized in a SQLite database for persistent storage and retrieval. Custom Python3 scripts were developed for searching the database.

After extracting data on the presence of proteins, a Pandas DataFrame is created containing binary vectors of proteins belonging to the corresponding orthogroups. Binary vectors are sorted using hierarchical clustering. For each orthogroup that has missing elements, a search for potential homologs is conducted against the NCBI nonredundant protein sequence database (nr) using the blastp algorithm to complement the OrthoDB data.

**Figure 1.**
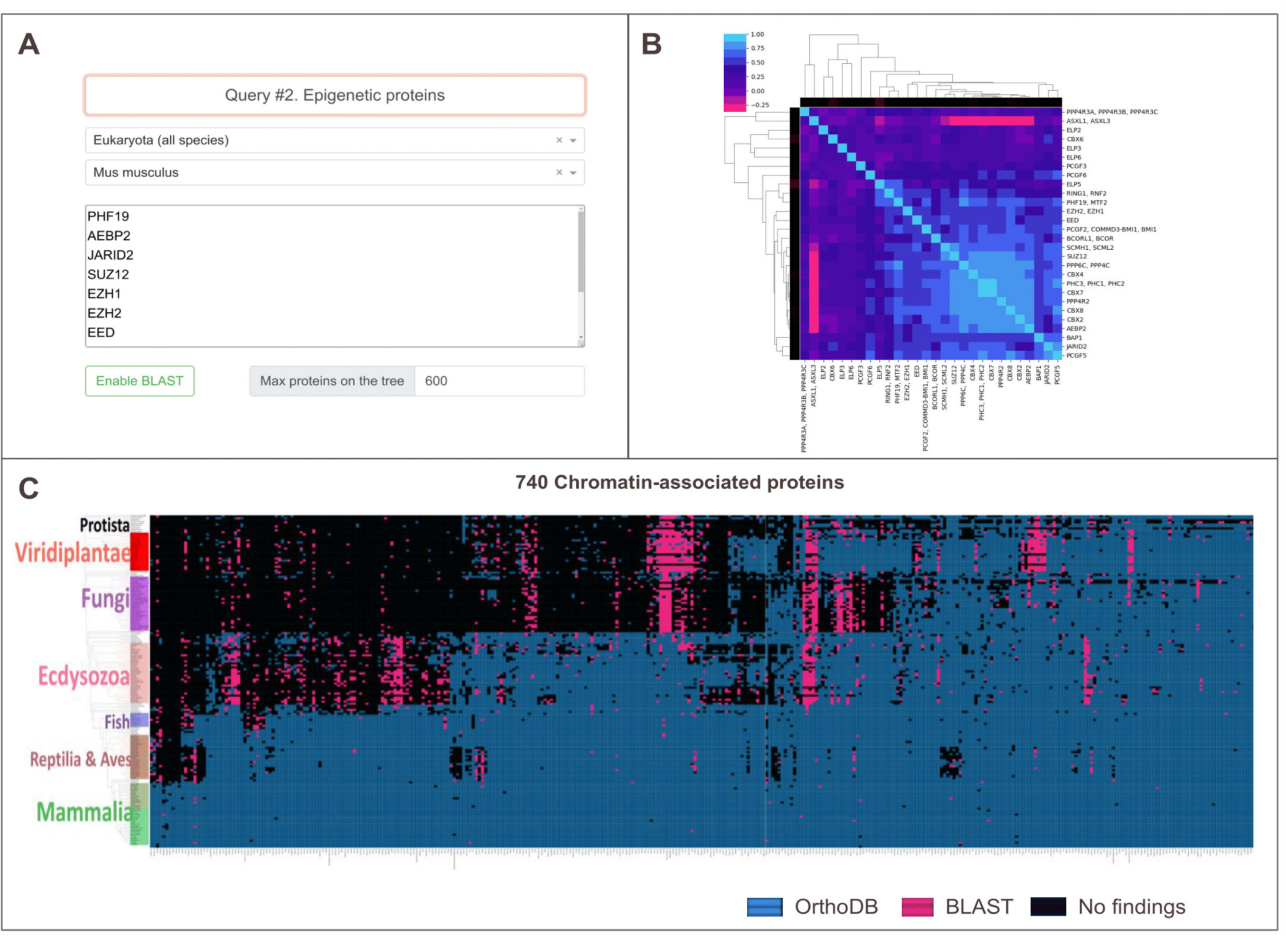
(**A**) Input area of the OrthoQuantum web server. Users can select target species by using the series of presented dropdown menus. (**B**) Matrix of Pearson correlation coefficients between orthogroups. (**C**) Phylogenetic profile of proteins associated with chromatin supplemented by BLAST search (*E*-value = 1e–10). The rows show 150 species of eukaryotes evenly covering the evolutionary tree. The columns are orthologous groups with 510 groups in total. Hierarchical clustering is applied only to the columns. Cells where the protein was found in OrthoDB are shown in blue, and BLAST findings are shown in purple.

According to the binary vectors, an orthogroup correlation matrix is constructed, which makes it possible to distinguish several clusters and subclusters (Figure 1 B). Proteins that function together, such as members of the same pathway or protein complex, often show similar patterns of conservation across phylogenetic clades. Proteins with similar conservation patterns will be in the same subcluster on the matrix, which might indicate that they are functionally linked.

### Input

Available eukaryotic species are divided into taxonomic categories based on levels of orthology: *Eukaryota*, *Metazoa*, *Viridiplantae*, *Vertebrata*, etc., because clustering of homologous sequences in OrthoDB occurs at the specified taxonomic level. Users can select sets of target species by using the series of presented drop down menus (Figure 1A). Once the target species have been selected, the user can input the sequence IDs in the textbox. Queries can be formulated using synonymous gene/protein names, identifiers, annotation keywords. If a certain entry yields multiple matching orthogroups the user is presented with a table that contains selectable rows with orthogroups. Corresponding graphs and data matching the selection condition are returned after the ‘Submit’ button is clicked. A search for potential homologs is performed using the blastp algorithm. Available parameters for the BLAST search are E-value threshold, sequence identity and query coverage.

Users then proceed to the visualization page. ‘My queries’ menu in the header allows the user to bookmark and access the results at a later time.

### Output

At this stage users are presented with a visualization of their results which features an orthogroup information table, heatmap of orthogroup correlations, and an interactive phylogenetic profile. If there is just one gene/orthogroup in the input, only the information table and phyloprofile will be shown.

#### Orthogroup table

**Figure 2.**
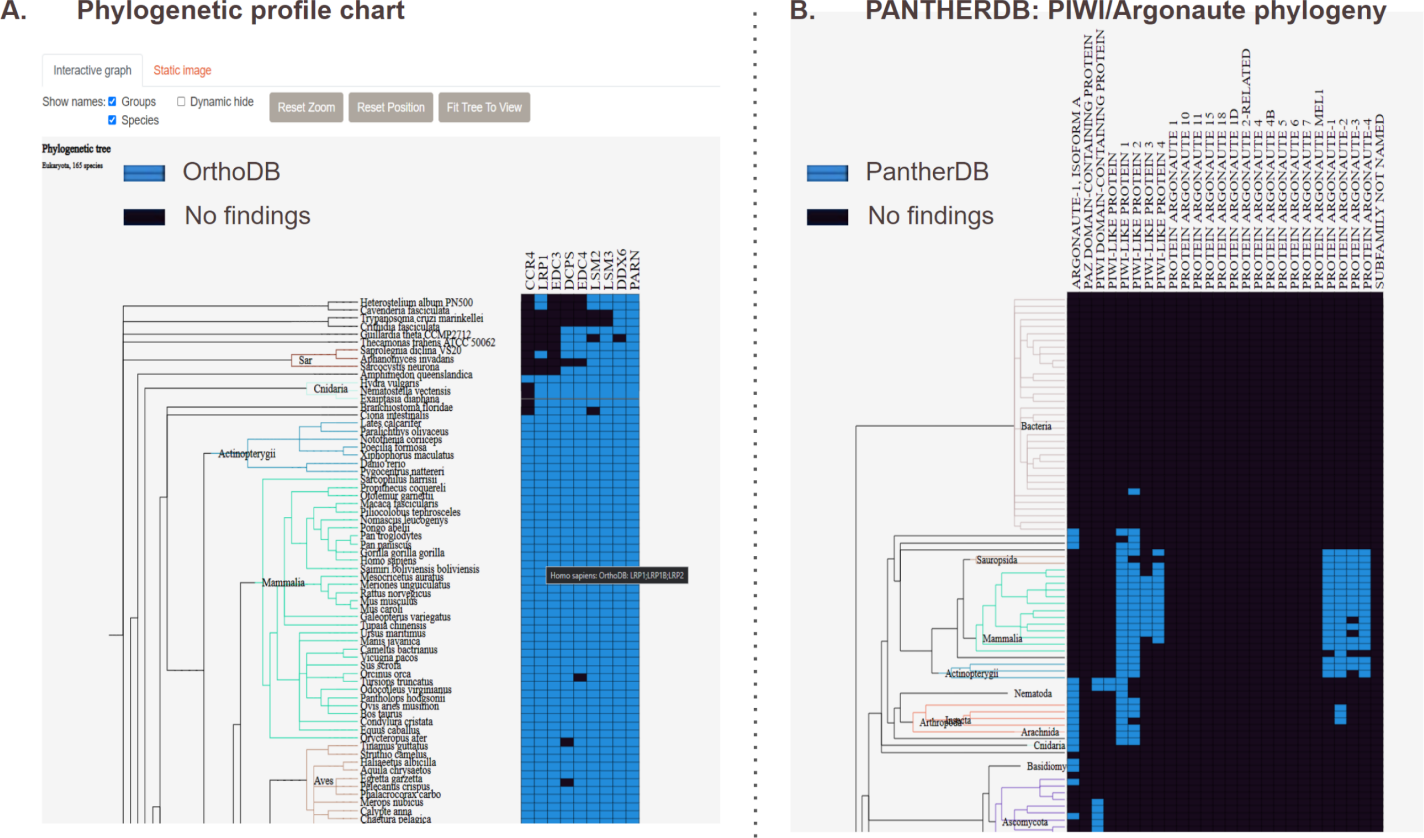
(**A**) Phylogenetic profile plot of nine epigenetic proteins in 165 eukaryotes. (**B**) PANTHERDB subfamily annotation for PIWI/Argonaute protein family.

A table of information related to each orthogroup is loaded that links query gene/protein names with orthogroup identifiers and contains some other useful information about the orthogroups, such as the evolutionary rate (‘evolrate’), median protein length. These annotations are computed by OrthoDB. The orthogroup IDs in the ‘OG label’ column link to the OrthoDB database from where the sequences can be downloaded directly in FASTA format. Clicking on the entries in the ‘Name’ column generates a phylogenetic profile of subfamilies of homologous proteins based on the PantherDB data in the new browser tab. This graph (Figure 2B) helps to analyze conservation profiles of proteins within orthologous groups that contain multiple paralogous genes.

#### Correlation matrix

The colors on the matrix (Figure 1B) reflect the values of the Pearson correlation coefficient. On both axes, a color bar is added corresponding to the percentage of homologs present in species: black corresponds to a high percentage and bright red corresponds to a low percentage. Ranked correlations between each pair of proteins with their percentiles are displayed in a table near the matrix.

#### Phylogenetic profile

On the bottom of the page a phylogenetic profile plot is constructed (Figures 1C, 2A). The columns show the orthogroup protein names, their order is defined by the hierarchical clustering. Clicking on the names opens the family/subfamily phylogenetic profile based on PantherDB annotation (Figure 2B). Rows of the heatmap show the eukaryotic genomes, major taxa on the species tree are labeled with different colors. The tree is zoomable. Mousing over an individual tile in the heatmap reveals the tooltip containing query species, gene names of both the query and orthologous genes. Clicking on it pins the tooltip and reveals detailed information, including the link to FASTA formatted sequences. The web server visualizes BLAST hits on the same tree. PhyD3 charts are rendered using SVG model, which is easy to use, but has limited performance. In our tests browsers can typically handle around 1000 orthogroup entries. The user can change the number of displayed proteins to improve performance. In this case, the proteins with the highest absolute values of correlation coefficient will be displayed.

## RESULTS

### Case studies

In order to illustrate the features of OrthoQuantum, we present case studies which show potential applications to similar problems.

### Presence/absence patterns of epigenetic factors

In the nucleus of eukaryotic cells, genomic DNA is associated with numerous protein complexes and RNA, forming a chromatin landscape. In this study, we assess the evolutionary relationships and repertoire of chromatin-associated proteins in eukaryotes. Epigenetic modifications are carried out by proteins, which can be subdivided into 3 groups: ‘writers’, ‘readers’ and ‘erasers’ of the epigenetic code (21). As the name suggests, ‘writers’ leave marks, ‘readers’ recognize them, and ‘erasers’ remove them. The coordinated action of these three groups determines the dynamic nature of epigenetic regulation. These and other epigenetic mechanisms involved in chromatin modification have been widely characterized in various eukaryotic species. We know much about the history of the system of DNA methyltransferases. Regarding many other components of epigenetic regulation, there is still little available information that has mostly been obtained experimentally for several model organisms. In this regard, interest lies in what interactions are lost or retained in other species.

Previously, the origin and coevolution of epigenetic proteins have been analyzed in a number of studies. Some studies focused on individual complexes and families of proteins including histones, INO80 and Snf2 complexes (22,23). In two other studies, researchers worked with a set of all identified epigenetic proteins from proteomes of model organisms. The approach in the first work was to compile a reference dataset of 110 Drosophila proteins and search for their homologs in prokaryotes (15 species) and eukaryotes (40 species) (24). The authors searched for homology with the local alignment method and in the MetaPhors database, which stores the calculated phylogenetic trees of orthologs and paralogs (25). In the second work, the InParanoid algorithm was used to predict the orthologs of 300 epigenetic proteins and to complement gene annotations in four model organisms (26).

Despite an increasing number of available genomes of nonmodel species from omics studies, the number of proteins and the number of taxa studied were limited in these studies. To investigate phylogenetic profiles of epigenetic proteins in eukaryotes we used dataset from the EpiFactors database (27) that includes 740 well-studied chromatin-associated proteins acting independently or as part of a multiprotein complex. Their UniProt AC identifiers were fed into the algorithm to construct a phylogenetic profile of proteins in 150 species from protozoa to mammals ordered according to their taxonomy from the NCBI Taxonomy database. Some proteins have a common origin; therefore, some groups contain several proteins at once, and there are 510 orthologous groups. As shown in the graph (Figure 1C), hierarchical clustering was applied to binary vectors. As a result, regions with different profiles of protein coverage became visible. An additional BLAST search with a threshold of 1e–8 made it possible to fill in many single gaps and, for some proteins, even entire columns. In total, 5% of the cells were filled. Several permutations have occurred; for example, more potential homologs appeared in *Ecdysozoa* species for such genes as Dapk3, Usp49, Ddx50, Chd7, Tssk6 and Pkn1.

Orthogroups on the right side of the graph were found in all eukaryotic species: Kat7—a histone acetyltransferase, Hdac1—a histone deacetylase, Smarcad1—protein of the SWI/SNF complex, Kdm5c—a lysine demethylase, Hat1—a histone acetyltransferase, Prmt6—an arginine N-methyltransferase, Rbbp4, Kat7, Prkaa1, Smarcad1, Ppp2ca, Cul4b, Msh6, Hdac1, Hdac4, Kdm5c, Hat1 and Prmt6. On the left edge, there are orthogroups found in mammals, amphibians, fishes. In birds and some reptiles, they are absent. This area contains proteins Daxx, Tfpt and Sp100. Proteins that only exist in mammals: Dzip3 – an ubiquitin ligase, Dppa3 – an early developmental protein responsible for imprinting, Sap25 and Rpr8.

We inspected the horizontal gaps more closely in the phylogenetic profile of 60 protein cofactors of histone modifications (Supplementary Figure S1). *Crithidia fasciculata*, *Trypanosoma cruzi*, *Vavraia culicus* and *Nosema ceranae* are very similar in the patterns of homolog absence. All of the listed species are parasitic and unicellular: the first pair are protozoans with intracellular and extracellular forms, and the second pair are obligate intracellular microsporidian parasites. Proteomes of such species have shrunk during evolution, and their minimal molecular repertoire seems to be allowed by an intimate relationship with the host cell. Earlier, with the help of our tool we discovered a reduction in the cellular machinery of piRNA biogenesis in the trematode *Schistosoma japonicum*, which is a multicellular parasite of mammals. By binding to the PIWI protein, piRNAs are able to limit the expression of retrotransposons at the posttranscriptional level using epigenetic silencing. Compared to other eukaryotes, Schistosoma does not have homologs of key enzymes Tdrd1, Tdrd9, Tdrkh, MitoPLD, MAELSTORM and Mov10L1 (Supplementary Figure S2). These proteins are involved in the biogenesis of not only piRNAs but also other small noncoding RNAs (siRNAs, miRNAs), which are normally expressed in schistosomes. Schistosomes may have alternative transposon silencing pathways that have not been studied to date.

### Evolution patterns of Polycomb proteins in plants

**Figure 3.**
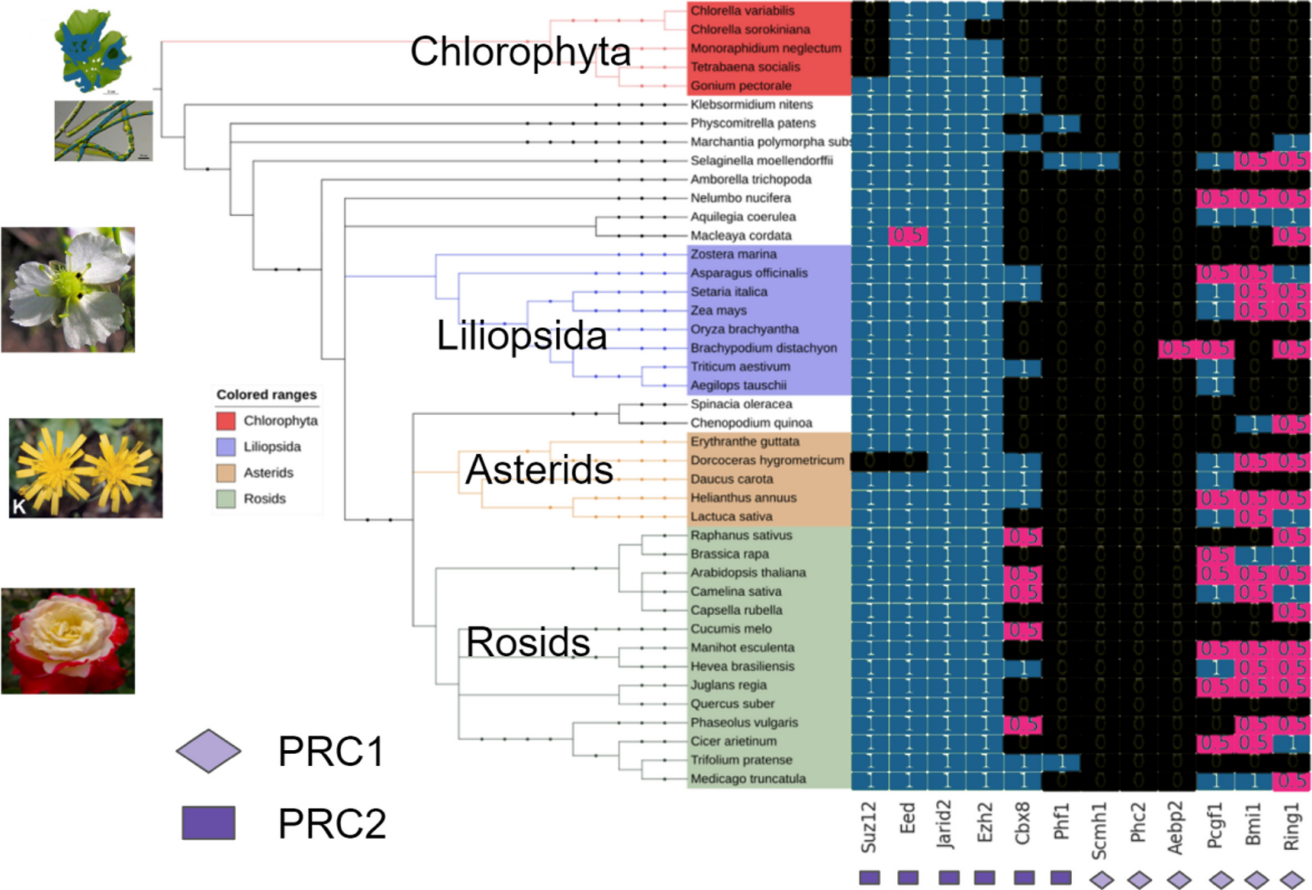
Phylogenetic profile of the PRC2 complex members (Suz12, Eed, Jarid2, Ezh2, Phf1 and Aebp2) and PRC1 (Phc2, Pcgf1, Bmi1, Ring1 and Scmh1) in plants. The presence and absence of orthologs in the database are indicated in blue and black, respectively, and the cells in which the findings were made using BLAST (*E*-value = 1e–6) are marked in purple.

In plants, PRC1 was considered absent until the homologous proteins of the RING zinc fingers in Arabidopsis were characterized (RING1a/b and BMI1). This complex is involved in a variety of processes during vegetative and reproductive development including the control of stem cell determination, flowering time, and seed development (28). On the presence plot at the *Viridiplantae* level (Figure 3), proteins of the PRC2 complex (Suz12, Eed, Jarid2 and Ezh2) are present in almost all species (except for chlorophytic algae, which do not have Suz12).

The fact that only a fraction of the proteins are found in OrthoDB may indicate that PRC1 complexes differ significantly between animals and plants. An additional BLAST search supplemented homologs of the Pcgf1, Bmi1 and Ring1 proteins in all clades, except for chlorophytic algae. Apparently, the proteins of the PRC1 complex have a greater evolutionary sequence divergence than the PRC2 proteins. The main components of PRC1, the RING proteins (Ring1 and Bmi1) and the protein with the chromo-domain LHP1, exist in the ancestor of seed plants and are involved in the formation of a functional complex with other subunits (29). These proteins form a complex similar in domain composition and function to animal PRC1 (30,31). LHP1 is mainly localized with H3K27 me3 in vivo and binds to trimethylated lysines of H3K27, suggesting that LHP1 is a functional homolog (32,33). The accompanying plant PRC1 factors include the proteins VRN, ING1/2, and VAL1. A phylogenetic profile was plotted for the specific Arabidopsis proteins but at the level of *Viridiplantae* (Supplementary Figure S3). Most plant species also have these proteins, some of which are found in chlorophyte algae. In the remaining gaps, no proteins were found using BLAST. Thus, the existing classification of epigenetic protein orthologs in databases such as OrthoDB can be complemented because the presence/absence of protein homologs sometimes does not correspond to the information given in the literature for certain species and taxa.

## DISCUSSION

Certain aspects of the phylogenetic profiles of proteins require interpretations and decisions by specialists. We therefore developed an automated procedure for searching and visualizing the phylogenetic distribution of proteins (orthologous groups) and their mutual correlation. The resulting web tool makes full use of homology data from OrthoDB, PantherDB and NCBI. Our study shows an example of using the tool for comprehensive examination of the evolution of chromatin-associated proteins. OrthoQuantum allows the user to submit protein queries, inspect the output in graphic format and download the output. Multiple researchers can easily access the tool. One of its strengths is the ability to choose between a compact set of species with a good quality of genome assembly and a full set of species that may provide better resolution for a phylogenetic profile in wide taxonomic groups such as Eukaryota. OrthoQuantum can display data from the set of over 1000 fully sequenced eukaryotic genomes and predicted orthologs.

The OrthoQuantum web server does not yet have the capability to implement whole proteome profile search for linkages. In the future updates we plan to choose a scoring metric and make pre-computed pairwise co-occurrence scores for all proteins at all levels of orthology. The profiles will be stored in the web server database.

Phylogenetic profiles are not very specific and, as a rule, do not provide information on the exact role of a protein in the process but are often decisive for further analysis. This study expanded the understanding of chromatin evolution by searching for homologs of 717 human epigenetic proteins in other eukaryotes. Epigenetic factors unique to animals (Tdrd3/7, Tdrkh, Gadd45 and Rybp), mammals (Dzip3, Dppa3 and Tex19) and plants (VRN2, ING1 and VAL1) have been identified. More research would be relevant to detect evolutionary events such as gene loss, duplications, and nonorthologous substitutions in the epigenetic repertoire.

Creation and analysis of the phylogenetic profiles and correlation matrices in 1200 eukaryotic species allowed us to draw the following three conclusions: (i) OrthoDB database sometimes has incomplete clusters of orthologs at the *Eukaryota* level: not all of the available homologs fall into orthologous groups of plants and protozoa, and gaps are observed in the clades that have homologs proven in studies; (ii) the proteins of the Polycomb PRC1 complex diverge significantly between animals and plants, while PRC2 is more conserved in all eukaryotes; (iii) the trematode *Schistosoma japonicum* completely lacks the biogenesis apparatus of piRNAs and the PIWI proteins themselves, which suggests an alternative pathway for transposon silencing.

## DATA AVAILABILITY

The OrthoQuantum v1.0 source code is open-source on GitHub (https://github.com/ilnitsky/OrthoQuantum_v1). The web server is available at http://orthoq.bioinf.fbb.msu.ru.

## Supplementary Material

gkac385_Supplemental_File
