# Supplementary material for: OrthoQuantum: visualizing evolutionary repertoire of eukaryotic proteins
Source: Nucleic Acids Res. 2022 May 24;50(W1):W534–40. doi: 10.1093/nar/gkac385 (PMC9252792; doi:10.1093/nar/gkac385)
Supplement: gkac385_Supplemental_File [file gkac385_supplemental_file.pdf]

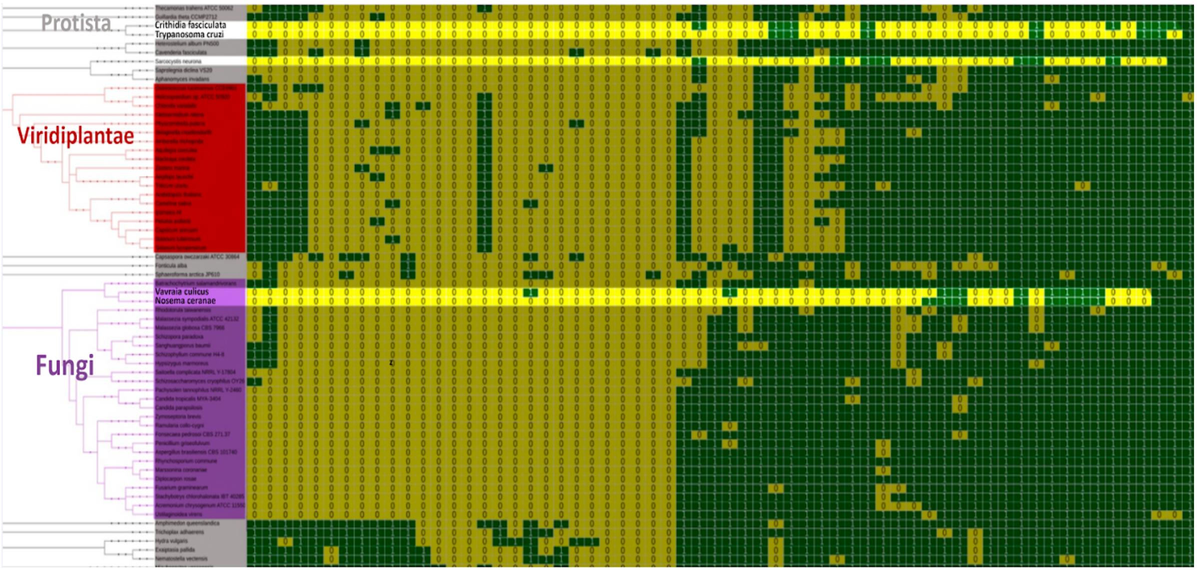

Supplementary Figure 1. Phylogenetic profile of proteins-cofactors of histone modifications. Organisms that differ from the main group are marked with a light stripe. The presence and absence of orthologs in the database are indicated in green and yellow, respectively.

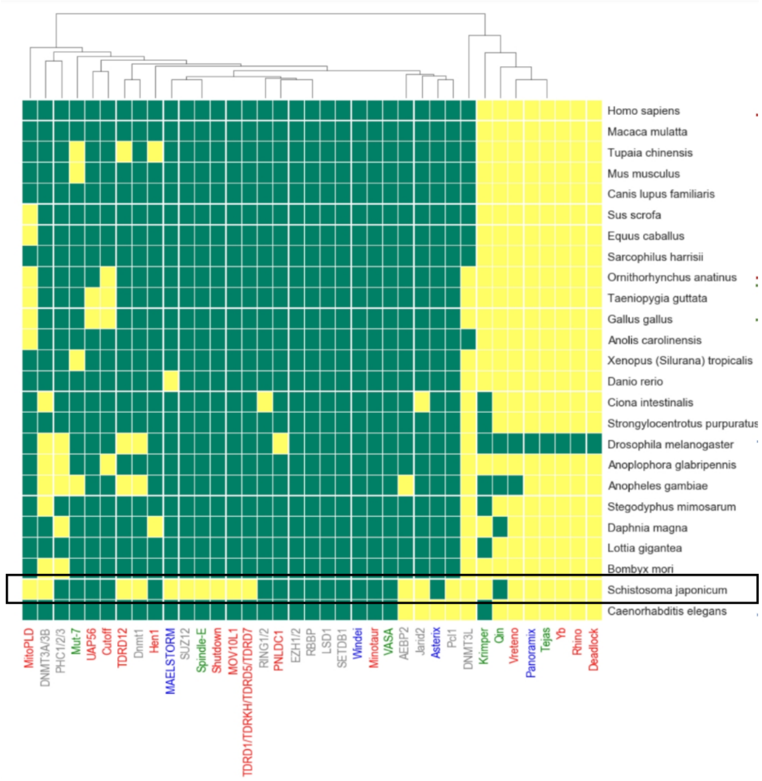

Supplementary Figure 2. Phylogenetic profile of proteins involved in biogenesis of piRNA. The presence and absence of orthologs in the database are indicated in green and yellow, respectively.

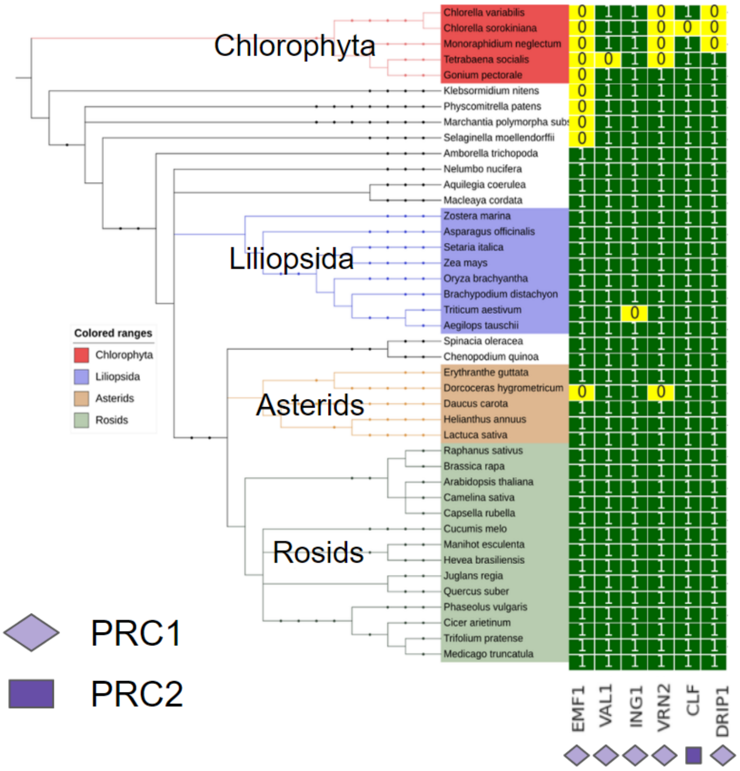

Supplementary Figure 3. Presence of proteins of the Polycomb group in *Viridiplantae* in 6 orthologous groups: EMF1, VAL1, VRN2, ING1, DRIP1 (PRC1), CLF (PRC2). The orthogroup DRIP1 includes homologous proteins RING1A, BMI1, LHP1. On the left are the color coding of the main taxa. The presence and absence of orthologs in the database are indicated in green and yellow, respectively.
